# Neoadjuvant study of niraparib in patients with HER2-negative, *BRCA*-mutated, resectable breast cancer

**DOI:** 10.1038/s43018-022-00400-2

**Published:** 2022-07-04

**Authors:** Laura M. Spring, Hyo Han, Minetta C. Liu, Erika Hamilton, Hanna Irie, Cesar A. Santa-Maria, James Reeves, Peng Pan, Ming Shan, Yongqiang Tang, Julie R. Graham, Sebastien Hazard, Leif W. Ellisen, Steven J. Isakoff

**Affiliations:** 1 Massachusetts General Hospital and Harvard Medical School, Boston, MA USA; 2 Moffitt Cancer Center-McKinley Outpatient Clinic, Tampa, FL USA; 3 Mayo Clinic, Rochester, MN USA; 4 Sarah Cannon Research Institute/Tennessee Oncology, Nashville, TN USA; 5 Icahn School of Medicine at Mount Sinai, New York, NY USA; 6 Sidney Kimmel Comprehensive Cancer Center at Johns Hopkins, Baltimore, MD USA; 7 Florida Cancer Specialists-South/Sarah Cannon Research Institute, Fort Myers, FL USA; 8 GSK, Waltham, MA USA; 9 Ludwig Center at Harvard, Boston, MA USA; 10 Present Address: EQRx, Cambridge, MA USA; 11 Present Address: Translational Discovery & Development, Boston Pharmaceuticals, Boston, MA USA; 12 Present Address: Alkermes Incorporated, Waltham, MA USA; 13 Present Address: Bicycle Therapeutics, Boston, MA USA

**Keywords:** Breast cancer, Targeted therapies, Cancer, Cancer therapy

## Abstract

This single-arm pilot study (NCT03329937) evaluated neoadjuvant niraparib antitumor activity and safety in patients with localized HER2-negative, *BRCA*-mutated breast cancer. Twenty-one patients received niraparib 200 mg once daily in 28-day cycles. After 2 cycles, tumor response (≥30% reduction from baseline) by MRI was 90.5% and 40.0% (6 of 15) of patients who received only niraparib (2–6 cycles) had pathological complete response; no new safety signals were identified. High niraparib intratumoral concentration was observed.

## Abstract (web summary)

Isakoff and colleagues demonstrate the safety and feasibility, as well as the clinical efficacy, of neoadjuvant niraparib in a phase II study in patients with HER2-negative and *BRCA*-altered breast cancer.

## Main

Neoadjuvant therapy for locally advanced breast cancer (BC) aims to downstage tumors and enable breast-conserving surgery^1^. Pathological complete response (pCR) is associated with lower recurrence rates than residual invasive cancer at surgery after neoadjuvant therapy^1^. Poly(ADP-ribose) polymerase (PARP) inhibitors provide new, effective treatment options for *BRCA1/2*-mutated advanced/metastatic breast cancer^2^ by targeting homologous recombination deficiency (HRd)^3^. Talazoparib and olaparib are approved for HER2-negative, germline *BRCA*-mutated (g*BRCA-*mut) metastatic BC^4,5^.

Niraparib is a PARP-1/2 inhibitor approved for recurrent or advanced ovarian cancers^6^. Preliminary pharmacokinetic data showed higher niraparib concentrations in tumors than in plasma, including in *BRCA-*mut, triple-negative breast cancer (TNBC) and *BRCA*-wild-type ovarian xenograft models^7–9^, which may facilitate primary tumor penetration in the neoadjuvant setting.

This pilot study (NCT03329937) explored the antitumor activity of neoadjuvant niraparib for localized HER2-negative, *BRCA-*mut BC and assessed niraparib concentration in tumor versus plasma. Duration of niraparib treatment beyond cycle 2 was determined by clinician decision and based on observed patient responses.

As of 30 June 2020, efficacy-evaluable (two or more cycles) and safety (one or more niraparib dose) populations included 21 of 24 enrolled patients with tumor *BRCA* mutations. One patient discontinued due to protocol noncompliance after completing two niraparib cycles. No patients received fewer than two cycles of niraparib, 19.0% received two cycles and 81.0% received more than two cycles. Six patients (28.6%) received post-niraparib neoadjuvant chemotherapy (NACT); all patients underwent surgery: 14 patients had *BRCA1*mut, 6 had *BRCA2*mut and 1 had *BRCA1/2*mut; 15 patients (71.4%) had TNBC and 6 patients (28.6%) had hormone-receptor positive (HR^+^) BC (Supplementary Table 1).

**Fig. 1:**
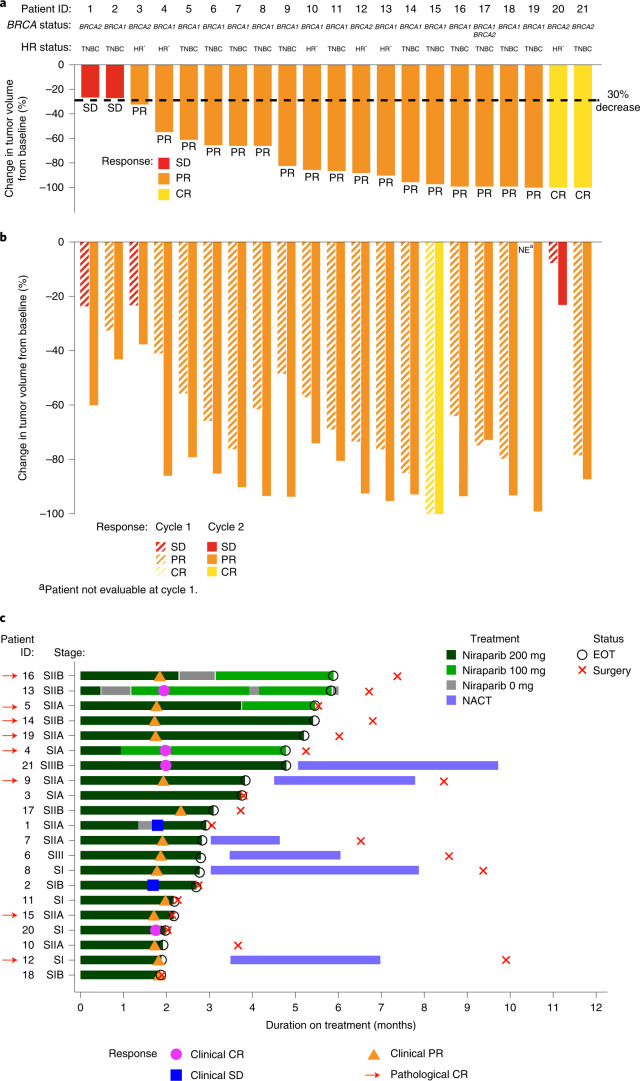
Clinical response and change in tumor volume by MRI and ultrasound, and clinical and pathological response patient journeys by MRI. **a**, Response by MRI at the end of cycle 2 of niraparib. **b**, Response by ultrasound after cycles 1 and 2. **c**, Presence of pCR, defined as *ypT0/Tis ypN0*, made at the time of surgery (*n* = 21 patients). EOT, end of treatment; NE, not evaluable; SI, stage I; SII, stage II; SIII; stage III. Source data

Tumor response by magnetic resonance imaging (MRI) after 2 cycles (primary endpoint) was 90.5% (95% confidence interval (CI): 69.6, 98.8%), with 2 CRs and 17 partial responses (PRs) (Fig. 1a) (86.7% in TNBC, 100% in HR^+^). By ultrasound, 81.0% (95% CI: 58.1, 94.6%) of tumors responded (1 CR, 16 PRs) after 1 cycle of niraparib and 95.2% (95% CI: 76.2, 99.9%) (1 CR, 19 PRs) responded after 2 cycles (Fig. 1b). Median (range) decrease in tumor volume after 2 cycles was 86.4% (26–100%) by MRI and 87.2% (23–100%) by ultrasound; best response by ultrasound (≥2 cycles) was a 92.5% (23–100%) decrease.

Overall, eight patients (38.1%; 95% CI: 18.1, 61.6%) had pCR after neoadjuvant niraparib (niraparib duration, 1.9–5.9 months) (Fig. 1c). Of 15 patients, 6 (40.0%; 95% CI: 16.3, 67.7%; 5 TNBC, 1 HR^+^) who received only niraparib for 2–6 cycles had pCR; 2 of 6 patients (33.3%; 95% CI: 4.3, 77.7%; 1 TNBC, 1 HR^+^) who received NACT after niraparib had pCR. Six patients with pCR had *BRCA1*mut; 2 had *BRCA2*mut. Of 15 patients 6 (40.0%; 95% CI: 16.3, 67.7%) with TNBC and 2/6 (33.3%; 95% CI: 4.3, 77.7%) with HR^+^ BC had pCR. A summary of patient response, tumor characteristics and niraparib exposure can be found in Supplementary Table 2.

Median (range) duration of niraparib exposure was 2.9 (1.8–5.9) months. Overall, 19 of 21 patients (90.5%) experienced any-grade, niraparib-related, treatment-emergent adverse events (TEAEs; Supplementary Table 3). Grade ≥3, niraparib-related TEAEs included anemia (*n* = 3), neutropenia (*n* = 2), decreased neutrophil count (*n* = 2), hypertension (*n* = 1) and thrombocytopenia (*n* = 1). Two patients (9.5%) had a niraparib-related serious adverse event (AE: 1 thrombocytopenia, 1 fetal ventricular septal defect (grade 2) in the fetus of a patient with ~3 weeks’ niraparib exposure during pregnancy identified at the end-of-treatment visit). TEAEs led to niraparib dose reduction in 4 patients (19.0%; neutropenia, *n* = 1; thrombocytopenia, *n* = 1; neutrophil count decreased, *n* = 2). No patients discontinued treatment due to TEAEs and there were no deaths during the study.

**Fig. 2:**
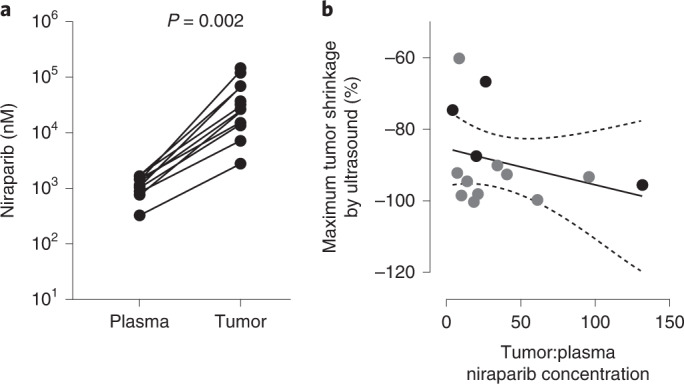
Niraparib concentration in plasma and tumor and association between reduction in tumor volume and tumor:plasma niraparib concentration. **a**, Niraparib concentration in patient plasma and tumor samples collected at the end of cycle 2 of niraparib, day 28 (*n* = 10 patients with time-matched samples; two-sided Wilcoxon’s matched-pairs, signed-rank test, *P* = 0.002). One patient in the analysis had a dose reduction to 100 mg before the end of cycle 2. **b**, Maximum tumor volume reduction based on ultrasound measurement after ≥2 months of niraparib treatment (maximal tumor reduction was −100%) and the fold difference in tumor versus matched plasma niraparib concentration (where available; for patients without available matched plasma samples, the plasma niraparib *C*_max_ value from C2D1 was used instead to estimate the fold difference), using a linear regression model *R*^2^ = 0.088; Spearman’s rank correlation (*ρ* = −0.26, two-sided *P* = 0.36). The gray dot indicates patients with time-matched tumor and plasma samples (*n* = 10 patients) and the black dot patients without time-matched plasma samples (*n* = 4 patients), for whom fold difference in tumor versus plasma niraparib concentration was estimated based on the plasma *C*_max_. The dashed lines indicate 95% CIs. Source data

**Extended Data Fig. 1:**
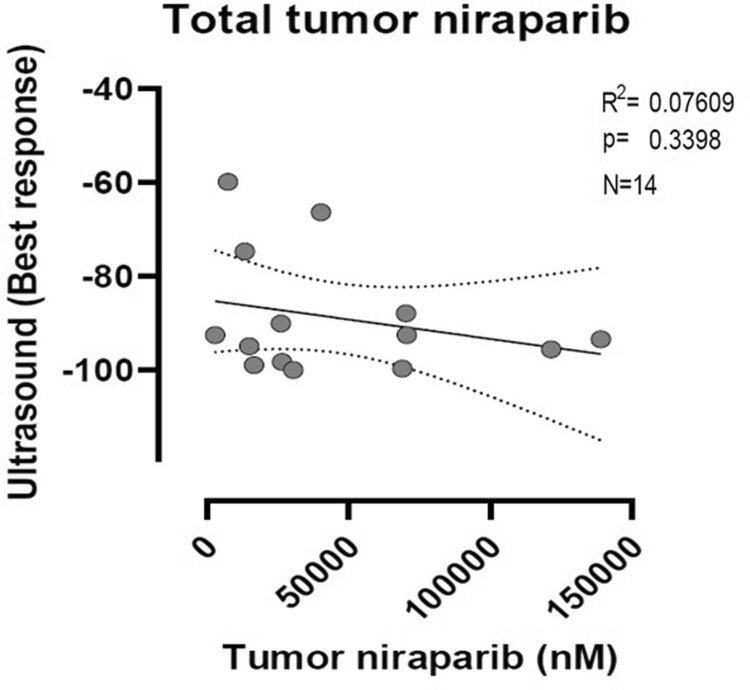
Association between reduction in tumor volume and total tumor niraparib concentration. Maximum tumor volume reduction based on ultrasound measurement after ≥2 months of niraparib treatment (maximal tumor reduction was −100%) and the fold difference in tumor versus total tumor niraparib concentration using a linear regression model *R*^2^ = 0.076; *P* = 0.34.•indicate patients with time-matched tumor and plasma samples (*n* = 14 patients) Dashed lines indicate 95% confidence intervals. *C*_max_, maximum concentration. Source data

In 10 patients with time-matched plasma/tumor samples collected after 2 cycles, mean (±s.d.) intratumoral niraparib concentrations were 35.2 ± 37.2-fold higher versus plasma (Wilcoxon’s matched-pairs signed ranks test, *P* = 0.002; Fig. 2a). A post-hoc analysis of the association of tumor:plasma niraparib concentration and tumor response was assessed by linear regression (Fig. 2b; *R*^2^ = 0.088; Spearman’s rank correlation *ρ* = −0.26, two-sided *P* = 0.36) including 95% confidence bands of best fit. Other parameters analyzed included total tumor niraparib concentration, which demonstrated a similar trend but was not statistically significant (Extended Data Fig. 1). However, due to the small sample size (*n* = 14), conclusive statements cannot be drawn from these data.

Neoadjuvant niraparib was highly active in patients with localized HER2-negative, *BRCA-*mut BC. There were no new safety signals and no discontinuations due to TEAEs.

After 2 cycles, >90% of patients experienced a clinical response; 38% had pCR after neoadjuvant niraparib, most of whom received only niraparib. Intratumoral niraparib concentrations were >30-fold higher than in plasma. Tumor penetration may be associated with reduced tumor volume, warranting further investigation. This is consistent with preclinical data showing superior tumor penetration by niraparib (3.3-fold higher exposure than plasma) versus other PARP inhibitors (for example, olaparib: 0.6- to 0.7-fold plasma concentration)^7^. In addition, niraparib concentrates in tumor and other tissues rather than circulating in the plasma; dose-normalized niraparib exposure was 10-, 51- and 100-fold higher versus olaparib in plasma, tumor and brain, respectively^7^. This, combined with the low clearance and high volume of distribution of niraparib, further supports a higher tendency of niraparib to concentrate in the peripheral body compartment and solid tumors, rather than in plasma^7^.

A phase II pilot study of neoadjuvant talazoparib also demonstrated clinical activity. All patients with g*BRCA-*mut BC received 6 months of neoadjuvant talazoparib; 53% (10/19) had pCR (primary endpoint) and 9 patients had dose reductions due to TEAEs^5^.

In our study, physicians could make treatment decisions based on observed responses at the end of cycle 2 by MRI or ultrasound, before receipt of additional therapy. Of 15 patients, 6 (40.0%) who received niraparib only (no NACT) had pCR; these patients received 2–6 cycles of niraparib. Given that five of the six patients achieving pCR in our study received four or more cycles of niraparib (no NACT), the rate of pCR achieved in this population is consistent with that of the neoadjuvant talazoparib study^5^. Furthermore, the INFORM trial reported that 18–26% of patients with stage I–III, *BRCA-*mut, HER2-negative BC had pCR with NACT (cisplatin or doxorubicin–cyclophosphamide)^10^. These promising results, determined from imaging and pCR rates, highlight the efficacy of neoadjuvant niraparib in *BRCA-*mut BC and support the use of pCR as a primary endpoint for future studies using niraparib. In addition, these results also suggest that chemotherapy use could potentially be de-escalated, reducing toxicity.

Sensitivity to PARP inhibitors has also been shown in somatic *BRCA-*mut ovarian cancer and in patients with mutations in other HRd-related genes^11^. Up to 69% of patients with TNBC have HRd and *PALB2* mutations are also associated with HRd^12^. A phase II trial of olaparib showed antitumor activity in metastatic BC with somatic *BRCA1/2* and germline *PALB2* mutations^13^. In addition, a phase II study of talazoparib monotherapy demonstrated activity of PARP inhibitors in patients with advanced HER2-negative BC and a HR pathway gene mutation, beyond *BRCA1/2*. RECIST response was seen in 3 of 12 BC patients who had a RECIST response (objective response rate 25%; 2 g*PALB2*, 1 g*CHEK2*/g*FANCA*/*sPTEN*) and 3 additional patients *(gPALB2*, *sATR*, *sPTEN)* had stable disease (SD) for ≥6 months^14^. Further investigations may identify additional genetic subgroups that are likely to respond to PARP inhibitors. Limitations of our study included small sample size and heterogeneity in treatment after neoadjuvant niraparib and the number of cycles of niraparib, limiting conclusions about pCR. However, this targeted, chemotherapy-sparing approach showed favorable pCR rates and tolerability, supporting future investigations.

In this pilot study, single-agent neoadjuvant niraparib demonstrated promising antitumor activity and high levels of tumor penetration in HER2-negative, *BRCA-*mut, localized BC. No new safety signals were identified.

## Methods

The study was conducted in accordance with the Declaration of Helsinki and good clinical practice guidelines following approval by ethics committees and institutional review boards at each study site (Moffitt Cancer Center, Tampa, FL; Mayo Clinic Rochester, Rochester, MN; Sarah Cannon Research Institute/Tennessee Oncology, Nashville, TN; Icahn School of Medicine at Mount Sinai, New York, NY; Sidney Kimmel Comprehensive Cancer Center at Johns Hopkins, Baltimore, MD; Florida Cancer Specialists-South, Fort Myers, FL; Pacific Shores Medical Group, Long Beach, CA; Memorial Health Care System, Hollywood, FL; Baylor College of Medicine, Houston, TX; Providence Portland Medical Center, Portland, OR; and Massachusetts General Hospital, Boston, MA). All patients provided written informed consent.

The first subject was enrolled on 12 April 2018 and the last on 15 May 2019. All 24 patients were recruited from 7 of 11 active sites (site 1: 3 patients; site 2: 5 patients; site 3: 2 patients; site 4: 6 patients; site 5: 5 patients; site 6: 2 patients; and site 7: 1 patient). Eligible patients were female or male adults with: primary operable, histologically confirmed, HER2-negative, localized BC; deleterious/suspected deleterious *BRCA1/2* mutations (germline, may include somatic); primary tumor size ≥1 cm; and Eastern Cooperative Oncology Group performance status 0–1. Patients were excluded for previous therapy for current malignancy, previous PARP inhibitor use or distant metastases.

Niraparib 200 mg orally once daily was given in 28-day cycles. This dose was chosen to reduce the likelihood of dose interruptions due to AEs, which predominantly occurred within cycles 1–3 in a previous study^15^. Patients with progressive disease (increase in tumor volume >20% per ultrasound) after cycle 1 discontinued; patients with CR, PR or SD continued into cycle 2. The primary endpoint was tumor response rate (change in tumor volume by breast MRI by investigator after two cycles). A clinical response was defined as ≥30% reduction in tumor volume from baseline without new lesions (≥PR). After cycle 2, patients proceeded directly to surgery, received NACT and then surgery, or received up to 6 cycles of niraparib before surgery with or without subsequent NACT, at the physician’s discretion.

Secondary endpoints were tumor response rate by breast ultrasound (≥30% reduction in tumor volume from baseline), change in tumor volume from baseline after cycle 2 by MRI and ultrasound, pCR at time of surgery (*ypT0/Tis ypN0* by American Joint Committee on Cancer staging v.7.0) and safety/tolerability until 30 d after last niraparib dose. Niraparib intratumoral and plasma concentrations (via qualified liquid chromatography–tandem mass spectrometry at cycle 2) were exploratory endpoints.

Tumor volume was calculated as (length × width × height × π)/6 (ref. ^16^). If too small to measure, change from baseline was imputed as 99%. TEAEs were graded using Common Terminology Criteria for Adverse Events v.4.03. Differences between plasma and tumor niraparib concentrations were assessed using Wilcoxon’s matched-pair, signed-rank test (significance level *P* < 0.05). Maximum concentration (*C*_max_) was used to estimate niraparib tumor/plasma ratio when time-matched plasma samples were missing. Linear regression (GraphPad Prism v.8.0) assessed the correlation between response and niraparib tumor:plasma ratio. Spearman’s rank correlation was also performed.

### Statistics and reproducibility

All statistical analyses were performed using SAS statistical software v.9.3 or later unless otherwise noted; data distribution was assumed to be normal, but this was not formally tested. No statistical methods were used to predetermine sample sizes, but our sample sizes are similar to those reported in previous publications^17^. Data collection and analysis were not performed blind to the conditions of the experiments. Clinical exclusion criteria were pre-specified and patients were not eligible for the study if any of these were met; no data points were excluded from the analyses.

### Reporting summary

Further information on research design is available in the Nature Research Reporting Summary linked to this article.

## Supplementary information

Supplementary Information Supplementary Tables 1–3.

Reporting summary

## Data Availability

GSK makes available anonymized individual participant data and associated documents from interventional clinical studies that evaluate medicines, on approval of proposals submitted to www.clinicalstudydatarequest.com and a data access agreement will be required. To access data for other types of GSK-sponsored research, for study documents without patient-level data, and for clinical studies not listed, please submit an inquiry via this website. Source data are provided with this paper.

## Source data

Source data Fig. 1 Raw data for Fig. 1.
Source data Fig. 2 Raw data for Fig. 2.
Source data Extended Data Fig. 1 Raw data for Extended Data Fig. 1.

## Extended data

are available for this paper at 10.1038/s43018-022-00400-2.
