# Author Correction: Neoadjuvant study of niraparib in patients with HER2-negative, *BRCA*-mutated, resectable breast cancer

**DOI:** 10.1038/s43018-022-00430-w

**Published:** 2022-08-05

**Authors:** Laura M. Spring, Hyo Han, Minetta C. Liu, Erika Hamilton, Hanna Irie, Cesar A. Santa-Maria, James Reeves, Peng Pan, Ming Shan, Yongqiang Tang, Julie R. Graham, Sebastien Hazard, Leif W. Ellisen, Steven J. Isakoff

**Affiliations:** 1 Massachusetts General Hospital and Harvard Medical School, Boston, MA USA; 2 Moffitt Cancer Center-McKinley Outpatient Clinic, Tampa, FL USA; 3 Mayo Clinic, Rochester, MN USA; 4 Sarah Cannon Research Institute/Tennessee Oncology, Nashville, TN USA; 5 Icahn School of Medicine at Mount Sinai, New York, NY USA; 6 Sidney Kimmel Comprehensive Cancer Center at Johns Hopkins, Baltimore, MD USA; 7 Florida Cancer Specialists-South/Sarah Cannon Research Institute, Fort Myers, FL USA; 8 GSK, Waltham, MA USA; 9 Ludwig Center at Harvard, Boston, MA USA; 10 Present Address: EQRx, Cambridge, MA USA; 11 Present Address: Translational Discovery & Development, Boston Pharmaceuticals, Boston, MA USA; 12 Present Address: Alkermes Incorporated, Waltham, MA USA; 13 Present Address: Bicycle Therapeutics, Boston, MA USA

**Keywords:** Breast cancer, Targeted therapies, Cancer, Cancer therapy

Correction to: *Nature Cancer* 10.1038/s43018-022-00400-2, published online 4 July 2022.

In the version of this article initially published, in the first sentence of the second paragraph now reading “Niraparib is a PARP-1/2 inhibitor approved for recurrent or advanced ovarian cancers,” the word “endometrial” originally appeared in place of “ovarian”. The error has been corrected in the HTML and PDF versions of the article.

