# Supplementary material for: Neoadjuvant study of niraparib in patients with HER2-negative, BRCA-mutated, resectable breast cancer
Source: Nat Cancer. 2022 Jul 4;3(8):927–31. doi: 10.1038/s43018-022-00400-2 (PMC9402431; doi:10.1038/s43018-022-00400-2)
Supplement: Supplementary file 1 — Supplementary Tables 1–3. [file 43018_2022_400_MOESM1_ESM.pdf]

nature portfolio

https://doi.org/10.1038/s43018-022-00400-2

Supplementary information

Neoadjuvant study of niraparib in patients with HER2-negative, *BRCA*-mutated, resectable breast cancer

In the format provided by the authors and unedited

Supplemental Tables

Supplementary Table 1. Baseline patient demographics and characteristics

Characteristic	Total (N=21)
Age, years	
Median (min, max)	43.0 (21, 73)
Age group, years, n (%)	
<65	19 (90.5)
≥65	2 (9.5)
Race, n (%)	
White	19 (90.5)
Black or African American	1 (4.8)
Asian	1 (4.8)
Body weight, kg	
Median (min, max)	68.0 (47, 110)
BMI, kg/m^2	
Median (min, max)	25.1 (18, 41)
ECOG PS score, n (%)	
0	20 (95.2)
1	1 (4.8)
Stage at initial diagnosis, n (%)	
I	8 (38.1)
II	10 (47.6)
III	3 (14.3)
Histologic type, n (%)	
Invasive ductal carcinoma	20 (95.2)
Invasive lobular carcinoma	1 (4.8)
BRCA mutation status, n (%)	
BRCA1	14 (66.7)
BRCA2	6 (28.6)
BRCA1 and *BRCA2*[a]	1 (4.8)
Hormone receptor status, n (%)	

TNBC	15 (71.4)
ER-positive and/or PR-positive disease	6 (28.6)

[a]One patient was positive for both *BRCA*1 and *BRCA*2 mutations.
BMI, body mass index; ECOG PS, Eastern Cooperative Oncology Group performance status; ER, estrogen receptor; PR, progesterone receptor; TNBC, triple-negative breast cancer

Supplementary Table 2. Summary of patient response, tumor characteristics and niraparib exposure

Patient ID	Tumor response*	*BRCA1/2*m status†	PrR/ER/HER2 status (+/−)	BC stage/ histologic grade at initial diagnosis	Duration of niraparib exposure (months)	NACT (post neoadjuvant niraparib, if received)
1	SD	*BRCA1* wt/ *BRCA2* deleterious	−/−/−	IIA/ G3	2.92	
2	SD	*BRCA1* deleterious/ *BRCA2* wt	−/−/−	IB/ G3	2.69	
3	PR	*BRCA1* wt/ *BRCA2* deleterious	−/+/−	IA/ G2	3.75	
4	PR	*BRCA1* deleterious/ *BRCA2* wt	+/+/−	IA/ G3	4.76	
5	PR	*BRCA1* deleterious/ *BRCA2* wt	−/−/−	IIA/ G3	5.45	
6	PR	*BRCA1* deleterious/ *BRCA2* wt	−/−/−	III/ G3	2.79	Carboplatin/taxol/ cyclophosphamide/doxorubicin
7	PR	*BRCA1* deleterious/ *BRCA2* wt	−/−/−	IIA/ G3	2.83	Carboplatin/paclitaxel
8	PR	*BRCA1* deleterious/ *BRCA2* wt	−/−/−	I/ G3	2.76	Carboplatin/paclitaxel/ cyclophosphamide/doxorubicin
9	PR	*BRCA1* deleterious/ *BRCA2* wt	−/−/−	IIA/ G3	3.81	Cyclophosphamide/doxorubicin
10	PR	*BRCA1* deleterious/ *BRCA2* wt	+/−/−	IIIA/ G3	1.91	
11	PR	*BRCA1* deleterious/ *BRCA2* wt	−/−/−	I/ G3	2.17	
12	PR	*BRCA1* wt/ *BRCA2* deleterious	+/−/−	I/ G3	1.87	Carboplatin/cyclophosphamide / doxorubicin/paclitaxel
13	PR	*BRCA1* deleterious/ *BRCA2* wt	+/−/−	IIB/ G3	5.85	
14	PR	*BRCA1* deleterious/ *BRCA2* wt	−/−/−	IIB/ G3	5.42	
15	PR	*BRCA1* wt/ *BRCA2* deleterious	−/−/−	IIA/ G2	2.17	
16	PR	*BRCA1* deleterious/ *BRCA2* wt	−/−/−	IIB/ G3	5.88	
17	PR	*BRCA1* deleterious/ *BRCA2* deleterious	−/−/−	IIB/ G2	3.09	
18	PR	*BRCA1* deleterious/ *BRCA2* wt	−/−/−	IB/ G3	1.84	
19	PR	*BRCA1* deleterious/ *BRCA2* wt	−/−/−	IIA/ G3	5.19	
20	CR	*BRCA1* wt/ *BRCA2* deleterious	+/+/−	I/ G2	1.97	
21	CR	*BRCA1* wt/ *BRCA2* deleterious	−/−/−	IIIB/ G3	4.76	Cyclophosphamide/taxol/doxor ubicin

*Tumor response by MRI at Cycle 2
†All are germline *BRCA1/2*m
BC, breast cancer; BRCA, breast cancer gene; CR, complete response; ER, estrogen receptor; HER2, human epidermal growth factor 2; m, mutation; NACT, neoadjuvant chemotherapy; PR, partial response; PrR, progesterone receptor; SD, stable disease; wt, wildtype; MRI, magnetic resonance imaging

Supplementary Table 3. Summary of most common niraparib-related TEAEs (any grade, reported in ≥10% patients)

Event, n (%)	Total (N=21)		
	Grade 1–2	Grade 3–4	Total Any Grade[a]
Any niraparib-related TEAE	12 (57.1)	7 (33.3)	19 (90.5)
Nausea	14 (66.7)	0	14 (66.7)
Fatigue	10 (47.6)	0	10 (47.6)
Anemia	2 (9.5)	3 (14.3)	5 (23.8)
Decreased appetite	4 (19.0)	0	4 (19.0)
Insomnia	4 (19.0)	0	4 (19.0)
Neutrophil count decreased	1 (4.8)	2 (9.5)	3 (14.3)
White blood cell count decreased	3 (14.3)	0	3 (14.3)

[a]No Grade 5 TEAEs were reported.
TEAE, treatment-emergent adverse event.
