# Supplementary material for: Neoadjuvant study of niraparib in patients with HER2-negative, BRCA-mutated, resectable breast cancer
Source: Nat Cancer. 2022 Jul 4;3(8):927–31. doi: 10.1038/s43018-022-00400-2 (PMC9402431; doi:10.1038/s43018-022-00400-2)
Supplement: Supplementary file 2 — Reporting summary [file 43018_2022_400_MOESM2_ESM.pdf]

nature portfolio

Corresponding author(s): Steven Isakoff

Last updated by author(s): May 5, 2022

Reporting Summary

Nature Portfolio wishes to improve the reproducibility of the work that we publish. This form provides structure for consistency and transparency in reporting. For further information on Nature Portfolio policies, see our Editorial Policies and the Editorial Policy Checklist.

Statistics

For all statistical analyses, confirm that the following items are present in the figure legend, table legend, main text, or Methods section.

n/a	Confirmed	
☐	☒	The exact sample size (*n*) for each experimental group/condition, given as a discrete number and unit of measurement
☐	☒	A statement on whether measurements were taken from distinct samples or whether the same sample was measured repeatedly
☐	☒	The statistical test(s) used AND whether they are one- or two-sided<br>*Only common tests should be described solely by name; describe more complex techniques in the Methods section.*
☒	☐	A description of all covariates tested
☒	☐	A description of any assumptions or corrections, such as tests of normality and adjustment for multiple comparisons
☐	☒	A full description of the statistical parameters including central tendency (e.g. means) or other basic estimates (e.g. regression coefficient) AND variation (e.g. standard deviation) or associated estimates of uncertainty (e.g. confidence intervals)
☐	☒	For null hypothesis testing, the test statistic (e.g. *F*, *t*, *r*) with confidence intervals, effect sizes, degrees of freedom and *P* value noted<br>*Give P values as exact values whenever suitable.*
☒	☐	For Bayesian analysis, information on the choice of priors and Markov chain Monte Carlo settings
☒	☐	For hierarchical and complex designs, identification of the appropriate level for tests and full reporting of outcomes
☒	☐	Estimates of effect sizes (e.g. Cohen's *d*, Pearson's *r*), indicating how they were calculated

Our web collection on statistics for biologists contains articles on many of the points above.

Software and code

Policy information about availability of computer code

Data collection	No custom software was used
Data analysis	GraphPad Prism V8.0; SAS statistical software version 9.3

For manuscripts utilizing custom algorithms or software that are central to the research but not yet described in published literature, software must be made available to editors and reviewers. We strongly encourage code deposition in a community repository (e.g. GitHub). See the Nature Portfolio guidelines for submitting code & software for further information.

Data

Policy information about availability of data

All manuscripts must include a data availability statement. This statement should provide the following information, where applicable:

- Accession codes, unique identifiers, or web links for publicly available datasets
- A description of any restrictions on data availability
- For clinical datasets or third party data, please ensure that the statement adheres to our policy

GlaxoSmithKline (GSK) makes available anonymized individual participant data and associated documents from interventional clinical studies that evaluate medicines, upon approval of proposals submitted to www.clinicalstudydatarequest.com. To access data for other types of GSK sponsored research, for study documents without patient-level data, and for clinical studies not listed, please submit an enquiry via this website.

Field-specific reporting

Please select the one below that is the best fit for your research. If you are not sure, read the appropriate sections before making your selection.

☒ Life sciences ☐ Behavioural & social sciences ☐ Ecological, evolutionary & environmental sciences

For a reference copy of the document with all sections, see nature.com/documents/nr-reporting-summary-flat.pdf

Life sciences study design

All studies must disclose on these points even when the disclosure is negative.

Sample size	This was a descriptive study, and no formal sample size calculations were performed; the sample size was determined for purposes of clinical considerations only and is similar to other published pilot studies. The sample size was also deemed sufficient for signal finding prior to initiating a larger study; it would provide approximately 80% power with 1 sided significance level of 0.15 to differentiate a response rate of 80% from a minimum response rate of 60%.
Data exclusions	Clinical exclusion criteria were pre-specified, and patients were not eligible for the study if any of these were met.
Replication	This was a single-arm, pilot study and so no formal replication of data was performed. The data acquired will be used to inform a larger clinical trial
Randomization	This is not relevant to our study as this was an open-label, single-arm pilot study with all participants receiving niraparib treatment.
Blinding	This is not relevant to our study as this was an open-label, single-arm pilot study with all participants receiving niraparib treatment.

Reporting for specific materials, systems and methods

We require information from authors about some types of materials, experimental systems and methods used in many studies. Here, indicate whether each material, system or method listed is relevant to your study. If you are not sure if a list item applies to your research, read the appropriate section before selecting a response.

Materials & experimental systems

n/a	Involved in the study	
☒	☐	Antibodies
☒	☐	Eukaryotic cell lines
☒	☐	Palaeontology and archaeology
☒	☐	Animals and other organisms
☐	☒	Human research participants
☐	☒	Clinical data
☒	☐	Dual use research of concern

Methods

n/a	Involved in the study	
☒	☐	ChIP-seq
☒	☐	Flow cytometry
☒	☐	MRI-based neuroimaging

Human research participants

Policy information about studies involving human research participants

Population characteristics	In the Safety Population, the median age for all participants was 43 years (range: 21 to 73 years), and 9.5% of participants were ≥65 years of age. Most participants (90.5%) were White and all were female. The median weight, height, and body mass index were 68.0 kg (range: 47 to 110 kg), 163.0 cm (range: 152 to 174 cm), and 25.1 kg/m2 (range: 18 to 41 kg/m2), respectively. The ECOG performance status at study entry was 0 for 95.2% of participants and 1 for 4.8% of participants. Median time from initial diagnosis to first dose was 1.38 years. The most frequently reported stage at initial diagnosis was Stage IIA cancer (28.6%), with the majority of participants (95.2%) diagnosed with invasive ductal carcinoma. Fourteen (66.7%) participants were positive for a BRCA1 deleterious mutation and 6 (28.6%) participants were positive for a BRCA2 deleterious mutation. One participant (4.8%) was positive for both BRCA1 and BRCA2 mutation status. All participants (100%) tested negative for HER2 status; of these, most of the participants tested negative for PR (16 [76.2%]) and ER (18 [85.7%]) status. The remaining participants were HER2-negative HR+ as follows: ER-positive (3 [14.3%] participants), PR-positive (5 [23.8%] participants), and both ER-positive and PR-positive (2 [9.5%] participants).The majority of participants (85.7%) had no prior anticancer treatment for nonprimary cancer.
Recruitment	Participants were recruited (between April 2018 and May 2019) by the Principal Investigators across 7 out of 11 active sites across the US. Written informed consent was obtained from each participant before enrollment according to the regulatory and legal requirements of the participating country. As part of this procedure, the Investigator explained orally and in writing the nature, duration, and purpose of the study and the action of the study drug in such a manner that the participant was aware of the potential risks, inconveniences, or adverse events (AEs) that could occur. The participant was informed that he/she was free to withdraw from the study at any time. The participant received all information that was required by regulatory

	authorities and ICH guidelines. The Investigator (or designee) provided the Sponsor with a copy of the IRB/IEC approved ICF prior to the start of the study.
Ethics oversight	Institutional Review Board (IRB)/Independent Ethics Committee (IEC) in accordance with local legal requirements for all study sites (Moffitt Cancer Center, Tampa, FL; Mayo Clinic Rochester, Rochester, MN; Sarah Cannon Research Institute/Tennessee Oncology, Nashville, TN; Icahn School of Medicine at Mount Sinai, New York, NY; Sidney Kimmel Comprehensive Cancer Center at Johns Hopkins, Baltimore, MD; Florida Cancer Specialists-South, Fort Myers, FL; Pacific Shores Medical Group, Long Beach, CA; Memorial Health Care System, Hollywood, FL; Baylor College of Medicine, Houston, TX; Providence Portland Medical Center, Portland, OR and Massachusetts General Hospital, Boston, MA)

Note that full information on the approval of the study protocol must also be provided in the manuscript.

Clinical data

Policy information about clinical studies

All manuscripts should comply with the ICMJE guidelines for publication of clinical research and a completed CONSORT checklist must be included with all submissions.

Clinical trial registration	NCT03329937
Study protocol	GlaxoSmithKline (GSK) makes available anonymized individual participant data and associated documents from interventional clinical studies that evaluate medicines, upon approval of proposals submitted to www.clinicalstudydatarequest.com. To access data for other types of GSK sponsored research, for study documents without patient-level data, and for clinical studies not listed, please submit an enquiry via this website.
Data collection	This study consisted of a Screening Period (Day -28 to Day -1), a Treatment Period, Presurgery chemotherapy (if appropriate), Surgery, a Safety Follow-up/End of Treatment (EOT) Visit occurring 30 days (+7 days) after the last dose of study drug, and an Off-Study Visit for the purposes of collecting the pathological complete response results for participants for whom the Safety Follow-up/EOT Visit occurred prior to surgery; for all other participants the Safety Follow-up/EOT Visit acted as the off-study visit. The expected treatment duration was approximately 56 days. Specifically, core biopsies occurred at screening and end of Cycle 2 (within 24 hours of the last dose of niraparib), tumor sample during surgery and blood samples were collected at screening, end of Cycle 1, Cycle 2 and pre-surgey.
Outcomes	Primary outcome: To evaluate the preliminary antitumor activity of niraparib assessed as the tumor response rate based on the change in tumor volume as measured by breast MRI, observed after treatment with niraparib in the neoadjuvant treatment of localized, human epidermal growth factor receptor 2 (HER2) negative, breast cancer susceptibility gene (BRCA) mutant breast cancer patients.<br>Secondary outcomes: To evaluate the preliminary antitumor activity of niraparib assessed by: presence of pathological complete response defined as ypT0/Tis ypN0 by receipt of pre-operative chemotherapy (Yes versus No), percentage change in tumor volume from baseline after 2 months of niraparib treatment. tumor response rate based on the change in tumor volume as measured by breast ultrasound, to evaluate safety and tolerability of niraparib per National Cancer Institute-Common Terminology Criteria for Adverse Events (NCI-CTCAE) v4.03 criteria
